# Underrepresentation of African-Americans in Alzheimer's Trials: A Call for Affirmative Action

**DOI:** 10.3389/fnagi.2016.00123

**Published:** 2016-06-03

**Authors:** Jaewook Shin, P. Murali Doraiswamy

**Affiliations:** ^1^Division of Translational Neuroscience, Department of Psychiatry, Duke University Medical CenterDurham, NC, USA; ^2^Duke Institute for Brain SciencesDurham, NC, USA; ^3^Duke Center for the Study of Aging and Human DevelopmentDurham, NC, USA

**Keywords:** African-Americans, Alzheimer's disease, recruitment issues, biomarkers, personalized medicine

Personalized medicine aims to tailor diagnosis and treatment based on an individual's personal genetic make-up and other predictive biomarkers—rather than a one-size fits all approach. African-Americans comprise 13.2% of the U.S. population (U.S. Census Bureau, 2014) and minorities (consisting of all but non-Hispanic Caucasian), now 37% of the U.S. population, are projected to reach closer to 57% in 2060 (U.S. Census Bureau, 2012). Indeed in coming decades minority population, including African-Americans, may exceed the Caucasian population in the U.S.

Dementia, especially Alzheimer's disease (AD), has emerged as one of the biggest threats to public health and personal wellbeing among older adults. Epidemiological studies, by nature of their community sampling, have been able to study risk for AD in racially representative populations. In such studies, older African-Americans have been reported as being more likely than older Caucasians to develop AD and other dementias (Gurland et al., 1999; Dilworth-Anderson et al., 2008; Potter et al., 2009; Barnes and Bennett, 2014; Alzheimer's Association, 2015). Potter et al. (2009), Gurland et al. (1999), and Barnes and Bennett (2014) report that prevalence of cognitive impairment or AD among African-Americans may be two or three times higher than in Caucasians. By 2050, according to The Alzheimer's Association annual report from 2010, proportion of racial minorities with AD will increase from 20 to 42%, with African-Americans increasing from 9 to 12% (Alzheimer's Association, 2010). A more recent 2015 report suggests that 16% of African-Americans were diagnosed with AD or other dementias compared to 8% of Caucasians (Alzheimer's Association, 2015).

## Current status

However, despite community studies suggesting they may be more susceptible to AD, African-Americans have been under-included in many prominent U.S. AD biomarker and clinical trials. In fact, barring a handful of studies in Asia and Africa, most of what we know about AD biomarkers and pathological changes comes almost exclusively from research studies of Caucasians (Brickman et al., 2008; Alzheimer's Association, 2010). Clinical trials and biomarker studies rely on convenience samples mostly recruited via advertisement. Further, we had great difficulty ascertaining the percentage of African-Americans in various trials since journals do not require trials to report a racial breakdown and many studies simply report percentage of Caucasians. Table 1 lists 10 major federally-funded biomarker studies conducted in the U.S. to illustrate that over half of these studies did not recruit adequate (approaching the USA national figure of 13%) numbers of African-American study subjects (for reasons specific to each study). For example, The Alzheimer's Disease Neuroimaging Initiative (ADNI) has made major contributions to our understanding of the pathological cascade and timeline of AD changes. Yet, ADNI-1 did not have sufficient number of African-Americans (< 5%) to reliably examine whether African-Americans differed from Caucasians. Likewise, with the exception of a multicenter trial of donepezil conducted exclusively in African-Americans (Griffith et al., 2006), almost all U.S. therapeutic information we have on what drugs work or do not work for AD (based on hundreds of industry sponsored AD clinical trials over four decades) is primarily derived from Caucasians.

**Table 1:** **Selected federally funded Alzheimer's biomarker and clinical research trials**.

**Study**	**Source**	**% AA (%)**	**Purpose of study**
ADNI-1	Weiner et al., 2013; Alzheimer's Disease Neuroimaging Initiative, 2015	4.8	To develop CSF, blood, and imaging biomarkers as outcome measures using a national cohort of CN, MCI, AD subjects
ADNIGO		3.1	To examine biomarkers in earlier stage of AD progression by including early MCI
ADNI-2		4.3	To develop CSF, blood, and imaging biomarkers as predictors of cognitive decline and outcome measures
DoD-ADNI	Sibener et al., 2014; Weiner, 2014	7.1	To use imaging and other biomarkers to establish the biological connections between TBI, PTSD and AD in Vietnam War veterans. Enrolment is still ongoing.
WHI-Cog	Goveas et al., 2011	7.0	To explore the causal relationship between depressive symptoms in postmenopausal women and MCI/dementia
SPRINT-MIND	Ambrosius et al., 2014	29.9^*^	To use cognitive assessments and structural brain imaging to measure the incidence of all-cause dementia in a subset of SPRINT participants
Cache County Study on Memory in Aging	Tschanz et al., 2013	<1^**^	To examine the role of genetic, psychosocial, and environmental risk factors for AD on its progression and late-life cognitive decline in a 12-year longitudinal study
Rush Memory and Aging Project	Bennett et al., 2012	<12.2^**^	To use CSF, blood, and postmortem brain neuropathologic evaluation to explore the relationship between age-related cognitive and motor decline and the risk of AD
WHICAP	Cosentino et al., 2010	31	To investigate the association between plasma amyloid beta and cognitive change and its implication for risk of AD
	Brickman et al., 2008	34.6	To use imaging biomarkers to compare brain region volumes among multiethnic cognitively normal elderly
Mayo Clinic Study of Aging	Roberts et al., 2008	<7.1^**^	To establish a population-based cohort for studying the prevalence, incidence, and risk factors of MCI and dementia

*%AA, percentage of African-American participants in each study; ADNI, Alzheimer's Disease Neuroimaging Initiative; GO, Grand Opportunities; CN, cognitively normal; MCI, mild cognitive impairment; AD, Alzheimer's disease; DoD, Department of Defense; TBI, Traumatic Brain Injury; PTSD, Post-traumatic Stress Disorder; WHI, Women's Health Initiative; SPRINT, Systolic Blood Pressure Intervention Trial; WHICAP, Washington Heights and Inwood Columbia Aging Project*.

* *The percentage of African-Americans refer to the sample demographics of SPRINT, the parent study of SPRINT-MIND*.

** *The percentage of African-Americans was not mentioned in the studies, and thus was deduced from the percentage of Caucasians; % Caucasian in Cache County Study on Memory and Aging was 99%, Rush Memory and Aging Project was 87.8%, and Mayo Clinic Study of Aging was 92.9%*.

Two major studies, SPRINT-MIND and the Washington Heights and Inwood Columbia Aging Project (WHICAP) did enroll sufficient numbers of African-Americans (Brickman et al., 2008; Cosentino et al., 2010; Ambrosius et al., 2014). Another study that had a substantive proportion of African-American participants was that conducted by Cosentino et al. (2010) as a part of WHICAP. Their goal was to associate plasma amyloid beta level with cognitive change indicative of AD progression, and in doing so, they recruited same number of Caucasian and African-American participants, each constituting 31% of the entire sample. They concluded that higher plasma amyloid beta level correlated with faster cognitive decline, noted in AD onset. As stated previously, to our knowledge, there has been only one US multicenter therapeutic drug trial done focused solely on African Americans with AD (Griffith et al., 2006). This 12-week multicenter trial of donepezil in 126 African Americans concluded it was safe and effective in mild-moderate AD (Griffith et al., 2006). However, the open label design (without placebo control) and the fact that ~51% of the subjects suffered adverse effects including diarrhea, hypertension and urinary tract infections, leaves open the question of whether the drug was truly effective.

Thus, while much progress has been made in AD research, it is clear that much of our knowledge about AD pathogenesis still comes from studies of Caucasians. One obstacle for recruiting African-American subjects is distrust of research studies led by hospitals, universities, and clinics due to historical mistreatments (Diaz et al., 2008; Ballard et al., 2010; Byrd et al., 2011; Lang et al., 2013). In this regards, it is encouraging to note that a recent survey of 5979 people in five US cities (Pease, 2013) found that 91% of African-Americans indicated interest in participating in research—suggesting that the onus is now on researchers to reach out to them and to not abuse their trust.

## Recommendations for future

While punitive approaches to enhance recruitment (e.g., tying grant payments to minority recruitment success or imposition of institutional penalties for failure to recruit sufficient minorities) may force change, we believe they are not ideal. We have some recommendations for enhancing African-American recruitment: (1) building long-standing partnerships with local African-American community organizations such as churches and initiations of forums, retreats, and other social events for greater approachability and getting to know the subjects on a more personal level (Ballard et al., 2010); (2) including a substantial budget for recruiting minorities in every major trial; (3) creating community based multiethnic registries (e.g., Brain Health Registry; Bryan ADRC Registry); (4) including a well powered hypothesis aimed at African Americans (or other major minority groups) in every major federally funded AD clinical trial; (5) explicit reporting in study reports and journal publications of percentage of African-Americans and other races, and whether race influenced any outcomes; (6) specific funding to conduct trials aimed at under-recruited minorities.

It is our hope that including a greater proportion of African-Americans in AD clinical trials will allow researchers to produce more generalizable results and a better understanding of race and ethnicity-specific differences in AD pathophysiology. This awareness and knowledge would help clinicians improve therapeutic target responses in patients and optimize the delivery of personalized care for more individuals with AD. Similar efforts aimed at Asians and Hispanics are already underway in other countries, and together these studies will help us paint a fuller picture of AD.

## Author contributions

JS was involved with topic selection, conducted necessary background literature research, and drafted the opinion article. PD oversaw the overall process and guided JS in selecting the topic and editing the draft.

## Funding

JS was supported by the Wrenn Clinical Research Scholars program at Duke University.

### Conflict of interest statement

The authors declare that the research was conducted in the absence of any commercial or financial relationships that could be construed as a potential conflict of interest. The reviewer TR and handling Editor declared their shared affiliation, and the handling Editor states that the process nevertheless met the standards of a fair and objective review.
